# Vergleich verschiedener Erhebungsmethoden zur Erfassung von Kompetenzen im Bereich des naturwissenschaftlichen Messens bei Tests mit Realexperimenten

**DOI:** 10.1007/s40573-025-00184-9

**Published:** 2025-07-11

**Authors:** Livia Murer, Susanne Metzger, Andreas Vorholzer, Angela Bonetti, Christoph Gut

**Affiliations:** 1 Abteilung Fachdidaktische Forschung, Pädagogische Hochschule Zürich (PHZH), Lagerstrasse 2, 8090 Zürich, Schweiz; 2 Institut für Bildungswissenschaften (IBW), Universität Basel und Pädagogische Hochschule FHNW, Hofackerstrasse 30, 4132 Muttenz, Schweiz; 3 TUM School of Social Sciences and Technology, Technische Universität München, Arcisstraße 21, 80333 München, Deutschland; 4 Pädagogische Hochschule Zürich (PHZH), Lagerstrasse 2, 8090 Zürich, Schweiz

**Keywords:** Experimentelle Kompetenzen, Kompetenzen des naturwissenschaftlichen Messens, Vergleich von Testverfahren, Tests mit Realexperimenten, Protokolle, Videoaufnahmen, Interviews, Experimental competencies, Competencies of scientific measurement, Comparison of assessment methods, Hands-on competence tests, Protocols, Video recordings, Interviews

## Abstract

Der Aufbau experimenteller Kompetenzen ist ein wesentliches Ziel naturwissenschaftlicher Bildung, womit auch deren Diagnose in den Fokus rückt. Allgemein werden Tests mit Realexperimenten als genauste Möglichkeit zur Erfassung experimenteller Kompetenzen betrachtet. Dabei können die Kompetenzen anhand verschiedener Erhebungsmethoden, z. B. Schüler*innen-Protokolle, Videoaufnahmen während der Durchführung oder Interviews über die Experimente, erfasst werden. Bislang gibt es keine Studien, die diese Erhebungsmethoden systematisch vergleichen und somit Rückschlüsse darauf ermöglichen, inwiefern die genutzte Methode die Genauigkeit des Ergebnisses der Kompetenzerfassung beeinflusst. Solche Erkenntnisse sind jedoch zentral, um je nach Kontext und Ziel der Erfassung der Kompetenzen fundiert entscheiden zu können, welche Erhebungsmethode hinreichend genau und dennoch möglichst ökonomisch ist. Dieses Desiderat greift die vorliegende Studie auf und nimmt dabei exemplarisch Kompetenzen des naturwissenschaftlichen Messens von Schüler*innen der Sekundarstufe I in den Blick: Ausgehend von Schüler*innen-Protokollen als ökonomische Möglichkeit zur Kompetenzerfassung wurde untersucht, inwiefern durch die Hinzunahme weiterer Erhebungsmethoden die Genauigkeit des Ergebnisses der Kompetenzerfassung erhöht wird. Hierfür wurden die Ergebnisse der Kompetenzerfassung anhand von Schüler*innen-Protokollen, Schüler*innen-Protokollen und Videos, Schüler*innen-Protokollen und Interviews sowie einer Kombination aller drei Methoden bei 108 Aufgaben mit Realexperimenten zum Messen verglichen. Die Ergebnisse zeigen, dass durch zusätzliche Interviews die Messkompetenzen genauer erfasst werden können als ohne Interviews. Des Weiteren deuten die Ergebnisse darauf hin, dass zusätzliche Videoaufnahmen während der Durchführung keinen entscheidenden Vorteil bezüglich der Genauigkeit des Ergebnisses der Kompetenzerfassung bringen, insbesondere dann nicht, wenn auch zusätzliche Interviews durchgeführt wurden.

## Abstract (en)

The development of experimental competencies is an essential goal of science education, which also brings their diagnosis into focus. In general, hands-on competence tests are considered to be the most accurate way to assess experimental competencies. Thereby the competencies can be assessed using different methods of data collection, e.g. student protocols, video recordings during experimentation or interviews about the experiments. So far, there are no studies that systematically compare these methods and therefore allow to draw conclusions about the extent to which the method used influences the accuracy of the assessment result. However, such findings are crucial in order to be able to make a well-founded decision as to which method of data collection is sufficiently accurate and yet as economical as possible, depending on the context and goal of the assessment. The present study takes up this desideratum and examines exemplary competencies in scientific measurement of lower secondary school students: Based on student protocols as an economic way to assess competencies it was investigated to what extent the accuracy of the assessment result is increased by the addition of further methods. For this purpose, the assessment results were compared using student protocols, student protocols and videos, student protocols and interviews, as well as a combination of all three methods for 108 hands-on tasks about scientific measurement. The results show that the measurement competencies can be assessed much more accurately with additional interviews than without interviews. Furthermore, the results indicate that additional video recordings do not provide an advantage with regard to the accuracy of the assessment result, especially if additional interviews were conducted.

## Einleitung

Der Aufbau von Kompetenzen aus dem Bereich des Experimentierens ist ein wesentliches Ziel naturwissenschaftlicher Bildung (EDK 2011; KMK 2005, 2020). In gängigen Modellierungen umfassen experimentelle Kompetenzen die Kenntnisse, Fähigkeiten und Fertigkeiten, die zur sachangemessenen Planung, Durchführung und Auswertung von Experimenten erforderlich sind (Gut et al. 2014; Hammann et al. 2008; Kranz et al. 2022; Schecker et al. 2016; Schreiber et al. 2014; Vorholzer et al. 2016). Zur Erfassung dieser Kompetenzen wurden bereits eine Reihe unterschiedlicher Testverfahren entwickelt, erprobt und diskutiert, zum Beispiel schriftliche Testinstrumente oder der Einsatz von Realexperimenten. Als genaueste aber auch sehr aufwändige Verfahren werden Kompetenzmessungen mit Realexperimenten angesehen (Baxter und Shavelson 1994; Gut-Glanzmann 2012; Schreiber 2012; Schreiber und Gut 2022). Realexperimente bieten grundsätzlich eine Reihe von verschiedenen Möglichkeiten, um auf die experimentellen Kompetenzen von Schüler*innen zu schließen. Zu diesen Möglichkeiten gehören beispielsweise die Auswertung des Experimentierprozesses selbst, zum Beispiel mittels Beobachtung oder Videoaufzeichnung, oder die Auswertung von Schüler*innen-Protokollen (Baxter und Shavelson 1994; Gott und Duggan 2002; Gut-Glanzmann 2012). Die verschiedenen Möglichkeiten der Auswertung von Realexperimenten (im Folgenden als *Erhebungsmethoden* bezeichnet) unterscheiden sich einerseits deutlich in dem mit ihrer Umsetzung verbundenen Aufwand. Andererseits ist davon auszugehen, dass aufwändigere Erhebungsmethoden, wie beispielsweise Prozessanalysen, auch eine genauere Erfassung der experimentellen Kompetenzen ermöglichen. Mit einer genauen Kompetenzerfassung ist im Rahmen dieser Studie gemeint, dass die Erfassung valide Rückschlüsse auf die Dispositionen der Schüler*innen zulässt, das bedeutet, dass die auf der Basis der Erfassung angenommene Ausprägung der Disposition möglichst genau der (nicht messbaren) tatsächlichen Ausprägung entspricht (vgl. auch Blömeke et al. 2015 und Heidrich 2017). Die Wahl einer geeigneten Erhebungsmethode für Realexperimente muss somit in der Regel im Spannungsfeld zwischen Genauigkeit und Test- sowie Auswertungsökonomie getroffen werden. Vor diesem Hintergrund ist von großer Relevanz, präzise zu beschreiben respektive zu erfassen, welchen Mehrwert an Genauigkeit eine aufwändigere Erhebungsmethode bei Tests mit Realexperimenten bringt, um ein für einen gegebenen „Erhebungsanlass“ valides und möglichst ökonomisches Verfahren zu wählen. Ziel der hier vorgestellten Studie ist es, verschiedene Erhebungsmethoden bei Tests mit Realexperimenten bezüglich der Genauigkeit des Ergebnisses der Kompetenzerfassung zu vergleichen, um somit die empirische Entscheidungsgrundlage für die Auswahl von Testverfahren zu stärken. Der Vergleich wurde exemplarisch am Beispiel von Aufgaben mit Realexperimenten zum naturwissenschaftlichen Messen durchgeführt, da das Messen ein wesentlicher Bestandteil der Durchführung und Auswertung von Experimenten (und damit auch von experimentellen Kompetenzen) ist.

## Kompetenzen des naturwissenschaftlichen Messens

Das Messen ist ein wesentliches Element naturwissenschaftlich-experimenteller Erkenntnisgewinnung und neben dem Beobachten und Modellieren ein wesentlicher Zugang zur Erhebung von Daten. Insbesondere wenn aus einem Experiment auf eine physikalische Größe oder den Zusammenhang zwischen Größen geschlossen werden soll, spielt das Messen eine fundamentale Rolle. Diese Bedeutung zeigt sich in Modellierungen experimenteller Kompetenzen sowie in normativen Vorgaben für den naturwissenschaftlichen Unterricht, die Kompetenzen des Messens explizit ausweisen (Modellierungen z. B. Gut et al. 2014; Schreiber et al. 2014; Wellnitz und Mayer 2013; normative Vorgaben z. B. KMK 2020, 2024; NRC 2012). Auch wenn die Aufnahme von Messwerten nur ein Element eines experimentellen Erkenntnisprozesses ist, zeigt sich bei genauer Betrachtung, dass das Messen eine Vielzahl von Teilkompetenzen beinhalten kann (siehe z. B. Überblick in Heinicke 2012; Priemer und Hellwig 2018). Dazu gehört zum Beispiel das Aufnehmen einzelner Messwerte, was sowohl kognitive Fähigkeiten (z. B. zur Auswahl eines geeigneten Messinstruments) als auch motorische Fertigkeiten (z. B. zur richtigen Handhabung eines Messgeräts) umfassen kann (Gott et al. n.d.; Haag et al. 2018; Priemer und Hellwig 2018). Auch das Planen von Messreihen und das Nutzen geeigneter Messstrategien (z. B. Messwiederholungen) kann als Teil von Messkompetenzen betrachtet werden. Neben diesen eher auf die Planung und Durchführung von Messungen bezogenen Teilkompetenzen umfassen breite Modellierungen von Messkompetenzen beispielsweise auch die Unterscheidung zwischen Messunsicherheiten und Messabweichungen oder die Identifikation und Beschreibung verschiedener Ursachen von Messunsicherheiten (z. B. Gut et al. 2014; Priemer und Hellwig 2018; Schulz 2022). Darüber hinaus wird zum Teil auch die Auswertung von gemessenen Werten als Teil von Messkompetenzen aufgefasst (z. B. Umgang mit Ausreißern: Allie et al. 1998; Millar und Lubben 1996; Nutzung von linearer Regression: Garatt et al. 2000; Schulz 2022). Während in engen Modellierungen das Messen primär einen Bestandteil der Durchführung von Erkenntnisprozessen darstellt und insbesondere die Aufnahme von Messwerten umfasst (z. B. Schreiber et al. 2014), spielt es in eher breiten Modellierungen bei Planung, Durchführung und Auswertung von Erkenntnisprozessen eine wichtige Rolle (z. B. Gut et al. 2014; Priemer und Hellwig 2018; Schulz 2022). Messkompetenzen beziehungsweise Kompetenzen im Allgemeinen werden in den genannten Modellierungen typischerweise im Sinne domänenspezifischer *latenter Dispositionen* (insbesondere kognitive, z. B. Kenntnisse, Fähigkeiten und Fertigkeiten; vgl. Weinert 2001; s. a. Vorholzer und von Aufschnaiter 2020) verstanden, die für zielgerichtetes Handeln erforderlich sind. Angesichts der Vielfalt und des Umfangs von Modellierungen von Messkompetenzen ist es deshalb mit Blick auf die Entwicklung oder Auswahl eines Testinstruments von großer Bedeutung, präzise zu beschreiben, auf welche Fähigkeiten und konzeptuellen Kenntnisse (z. B. Regeln, Definitionen, Strategien) mit diesem Instrument genau Rückschlüsse gezogen werden sollen (Vorholzer et al. 2016; Vorholzer und von Aufschnaiter 2020). Die Fähigkeit „Messwerte sachangemessen darstellen“ kann zum Beispiel umfassen, dass bei Messwerten immer auch die zugehörige Maßeinheit angegeben wird. Zudem könnte berücksichtigt werden, ob die Messwerte mit der richtigen Anzahl an Nachkommastellen angegeben werden. In beiden Fällen geht es um die Fähigkeit, Messwerte sachangemessen darzustellen. Für kompetentes Handeln genügt es jedoch im ersten Fall, intuitiv verstanden zu haben, dass Messwerte immer mit einer Maßeinheit angegeben werden müssen. Im zweiten Fall muss darüber hinaus auch mindestens intuitiv verstanden sein, welche Aussage die Anzahl der angegebenen Nachkommastellen über die Genauigkeit einer Messung macht (geltende bzw. signifikante Stellen; z. B. Heinicke 2012; Hellwig 2012).

Der hier vorgestellten Studie liegt ein eher breites Begriffsverständnis von Messkompetenzen im Sinne latenter Dispositionen zugrunde; es werden sowohl die Fähigkeiten zur Durchführung als auch zur Planung und Auswertung einfacher Messungen in den Blick genommen (z. B. Metzger et al. 2014). Bei der Operationalisierung dieser Fähigkeiten wurden – neben der Angabe des Messergebnisses in einem definierten Toleranzbereich als indirekte Überprüfung der Messdurchführung – die folgenden fünf Konzepte (K) als relevant angenommen:

K1: Ein Messwert muss immer mit der zugehörigen Maßeinheit angegeben werden.
K2: Eine geplante Messung muss zur zugrundeliegenden Problemstellung passen.
K3: Je höher die Genauigkeit des verwendeten Messinstruments ist, desto geringer ist die Messunsicherheit (z. B. Gott et al. n.d.).
K4: Die Messunsicherheit kann durch das Durchführen von Messwiederholungen und anschließender Mittelwertbildung reduziert werden (z. B. Gott et al. n.d.; Heinicke 2012).
K5: Die Messunsicherheit kann durch Vergrößerung der Menge, mit der gemessen wird, reduziert werden (z. B. Suida und Grabowski 2012).

Neben den beiden eher basalen Konzepten (K1, K2) zur Planung beziehungsweise Durchführung einzelner Messungen wurden somit auch Konzepte zum Umgang mit Messunsicherheiten (K3, K4, K5) betrachtet, die in der Planung und Auswertung von Messungen relevant sind. Diesen Konzepten liegt zu Grunde, dass jede Messung mit einer Unsicherheit behaftet ist und der „wahre Wert“ einer Größe in der Regel nicht bestimmt werden kann. Jenseits dieser Einschränkung ist es das Ziel jeder Messung, die Messunsicherheiten so weit wie nötig zu reduzieren, um ein ausreichend verlässliches Messergebnis zu erzielen (z. B. Fairbrother und Hackling 1997; Heinicke 2012; Hellwig 2012). Grundsätzliche Ansätze zur Verringerung der Messunsicherheit umfassen neben der Wahl eines möglichst genauen Messinstruments (K3) unter anderem auch das Durchführen von Messwiederholungen (K4) oder die Mengenvergrößerung (K5). Aus der wiederholten Messung der gleichen Größe kann einerseits auf die Größe der Unsicherheit geschlossen werden, andererseits kann der Mittelwert dieser Messreihe als beste Näherung der gesuchten Größe angesehen werden (Heinicke 2012). Die Vergrößerung einer zu messenden Größe kann dazu beitragen, die Messunsicherheit zu reduzieren, da mit zunehmender Größe der Einfluss der maximalen Genauigkeit des genutzten Messinstruments abnimmt. Exemplarisch lässt sich dies an folgendem Beispiel verdeutlichen: Mit einer Stoppuhr, welche die Zeit auf ± *t* Sekunden genau messen kann, soll die Periodendauer *T* eines Fadenpendels bestimmt werden. Bei der Messung *einer* Periode ergibt sich eine relative Messunsicherheit von *t*/*T*. Werden stattdessen *zehn Perioden* gemessen und das Ergebnis anschließend durch zehn geteilt, beträgt die relative Unsicherheit nur noch *t*/10*T* und ist somit deutlich geringer.

## Erfassung von Kompetenzen beim naturwissenschaftlichen Messen

In der Literatur werden verschiedene Möglichkeiten diskutiert, um experimentelle Kompetenzen im Allgemeinen zu erheben, zum Beispiel Paper-Pencil-Tests, simulationsbasierte Tests (z. B. mittels interaktiver Bildschirmexperimente) oder Tests mit Realexperimenten (z. B. Baur 2015; Baxter und Shavelson 1994; Dickmann et al. 2014; Gut-Glanzmann 2012; Schreiber et al. 2014; Webb et al. 2000). Testverfahren, die auf Realexperimenten basieren, werden dabei in der Regel als „Goldstandard“ angesehen (z. B. Baxter und Shavelson 1994; Schreiber und Gut 2022), da sie neben der Erfassung von Kompetenzen zur Planung und Auswertung auch die Erfassung von Kompetenzen zur Durchführung ermöglichen. Auch wenn solche Testverfahren mit einem großen materiellen und personellen Aufwand verbunden sind, werden sie deshalb für eine ganzheitliche Erfassung von experimentellen Kompetenzen als unerlässlich angesehen (z. B. Schreiber et al. 2014). Die Wahl eines Testverfahrens ist auch mit der Frage verbunden, welches Verständnis des Kompetenzbegriffs der Erfassung zu Grunde liegt. Kompetenz kann einerseits als domänenspezifische *latente Dispositionen* (Fähigkeiten, Kenntnisse etc.) verstanden werden, auf deren Ausprägung mittels der in einem Testverfahren gezeigten Performanz – zum Beispiel der gewählten Antwort in einem Multiple-Choice Test, der formulierten Antwort in einer offenen Aufgabe oder dem Verhalten in einer Experimentiersituation – Rückschlüsse gezogen werden (Blömeke et al. 2015; Heidrich 2017). Andererseits kann Kompetenz auch als das in einer spezifischen Situation gezeigte *manifeste Verhalten *selbst gedeutet werden (Blömeke et al. 2015). Während es bei einem Verständnis von Kompetenz als latente Dispositionen bei der Wahl des Testverfahrens primär um die Frage geht, inwiefern das Verfahren valide Rückschlüsse auf die zugrundeliegenden Dispositionen zulässt, geht es bei einem Verständnis von Kompetenz als manifestes Verhalten bei der Wahl des Testverfahrens primär darum, dass die Testsituation der späteren Realsituation, in der kompetent gehandelt werden soll, in relevanten Aspekten möglichst ähnlich ist (Blömeke et al. 2015; s. a. Vorholzer und von Aufschnaiter 2020). Aus letzterer Perspektive sind Testverfahren mit Realexperimenten wohl in der Regel anderen Testverfahren vorzuziehen, weil sie der Realsituation am ähnlichsten sind. In der naturwissenschaftsdidaktischen Forschung zur Erfassung experimenteller Kompetenzen werden diese Kompetenzen jedoch häufig im Sinne von Dispositionen verstanden (siehe z. B. Überblick in Schreiber und Gut, 2022), sodass die Frage nach der Wahl eines geeigneten Testverfahrens einer differenzierteren Antwort bedarf.

Die Überlegungen zur Erfassung experimenteller Kompetenzen lassen sich grundsätzlich auch für das naturwissenschaftliche Messen als Facette des Experimentierens übertragen. Ähnlich wie bei der Erfassung von experimentellen Kompetenzen hängt auch bei der Erfassung von Kompetenzen des Messens (im Sinne latenter Dispositionen) die Eignung der Testart von den spezifischen Fähigkeiten und Konzepten ab, die erfasst werden sollen (z. B. Christoph et al. 2015; Hammann et al. 2008; Schreiber 2012). Beispielsweise erscheinen zur Erfassung von Kompetenzen im Bereich der richtigen Verwendung eines Messgeräts bei der Durchführung von Messungen (z. B. Ablesen der Temperatur auf einem analogen Thermometer unter Berücksichtigung des Blickwinkels) Realexperimente zielführend, während sich Facetten wie das Planen oder Auswerten einer Messung vermutlich auch mithilfe von Paper-Pencil-Tests erheben lassen. In ähnlicher Weise ist anzunehmen, dass Simulationen für ausgewählte Facetten von Messkompetenzen das Potenzial haben, Tests mit Realexperimenten zu substituieren (z. B. Dickmann 2016; Schreiber et al. 2014).

Insgesamt ist somit festzuhalten, dass Realexperimente als Testverfahren zur Erfassung von experimentellen Kompetenzen im Allgemeinen aber auch von Messkompetenzen eine wichtige Rolle spielen. Die Erfassung von Kompetenzen in Realexperimenten kann dabei mithilfe verschiedener Erhebungsmethoden realisiert werden (Übersicht über mögliche Erhebungsmethoden: Baxter und Shavelson 1994; Gott und Duggan 2002; Gut-Glanzmann 2012):

- durch Protokolle, welche die Schüler*innen während des Experimentierens/Messens erstellen. Hierbei werden die Ergebnisse und Handlungen selbstständig durch die Schüler*innen notiert, wobei die vorgegebene Struktur der Protokolle von stark angeleitet bis sehr offen variieren kann (z. B. Baxter und Shavelson 1994; Emden und Sumfleth 2012; Gott und Duggan 2002; Hild et al. 2017).
- durch gezieltes Beobachten der Schüler*innen während des Experimentierens/Messens oder nachträglich mithilfe von Videoaufnahmen. Hierbei können die Kompetenzen anhand der experimentellen Handlungen der Schüler*innen durch geschulte Rater*innen erfasst werden (z. B. Baxter und Shavelson 1994; Emden und Sumfleth 2012; Gott und Duggan 2002; Hild et al. 2017).
- durch verbale Erläuterungen der Schüler*innen zu ihren Handlungen. Neben dem Lauten Denken während des Experimentierens/Messens können auch anschließende verbale Erläuterungen genutzt werden, wobei in anschließenden Interviews oft Erinnerungshilfen (Stimulated Recall: Konrad 2020) eingesetzt werden (z. B. Hild et al. 2017).

Alle drei Erhebungsmethoden – Protokolle, Beobachtungen und verbale Erläuterungen – können grundsätzlich dazu genutzt werden, um auf Experimentier- beziehungsweise Messkompetenzen von Lernenden im Sinne von latenten Dispositionen zu schließen. *Protokolle* bieten eine zeitökonomische Möglichkeit zur Erfassung von experimentellen Kompetenzen und entsprechend auch von Messkompetenzen in Realexperimenten, die sich grundsätzlich auch für large-scale Assessments eignet. Allerdings gelingt nicht allen Schüler*innen das Führen eines Protokolls (z. B. Gott und Duggan 2002), sodass nicht in allen Fällen davon ausgegangen werden kann, dass die Schüler*innen wirklich das protokollieren, was sie gemacht haben (z. B. Gut-Glanzmann 2012; Hild et al. 2019). Die *Beobachtung* von Schüler*innen während des Experimentierens wird oft als Benchmark zur Erfassung von Kompetenzen bei Test mit Realexperimenten betrachtet (z. B. Baxter und Shavelson 1994; Gott und Duggan 2002; Gut-Glanzmann 2012). Ein Nachteil dieser Methode ist jedoch, dass sie sehr ressourcenintensiv ist und sich darum weder für large-scale Assessments noch für die Bewertung ganzer Klassen eignet (Emden und Sumfleth 2012; Gut-Glanzmann 2012). Auch *verbale Erläuterungen*, zum Beispiel durch Lautes Denken oder nachträglich durchgeführte Interviews, können einige Herausforderungen mit sich bringen. Dazu gehören beispielsweise die Ressourcenintensität, der unnatürliche Charakter des lauten Verbalisierens von Gedanken bei Einzelarbeit, das Antworten gemäß sozialer Erwünschtheit (Konrad 2020) oder das verfälschte Wiedergeben der Gedanken in nachträglichen Interviews, etwa durch Erinnerungslücken oder inzwischen hinzugewonnener Erkenntnisse (Ericsson und Simon 1980). Dennoch ist der Mehrwert verbaler Erläuterungen nicht zu vernachlässigen, denn sowohl Protokolle als auch Beobachtungen ermöglichen nur einen stark eingeschränkten Einblick in die Gedanken der Schüler*innen (z. B. Abrahams et al. 2013; Gott und Duggan 2002). Wenn Schüler*innen ein Konzept zwar grundsätzlich verstanden haben, aber beispielsweise durch Zeitmangel in der Experimentier- oder Messsituation anders handeln, kann das erst durch verbale Erläuterungen erfasst werden. Gleiches gilt für den Fall, dass Schüler*innen zwar das Richtige tun, dies aber aus den falschen Gründen (z. B. mehrmals messen, weil es der/die Tischnachbar*in auch gemacht hat oder weil eben noch Zeit war). In diesen Beispielen zeigt sich auch noch einmal deutlich der Unterschied zwischen einem Verständnis von Kompetenz als manifestes Verhalten und Kompetenz als latente Dispositionen. Aus der Perspektive manifesten Verhaltens wäre eine Person A, die aus den falschen Gründen mehrmals misst, kompetenter als eine Person B, die zwar um die Bedeutung des mehrmaligen Messens weiß, dies aber aus praktischen Gründen nicht tut, weil Person A in einer praxisnahen Situation das richtige Verhalten zeigt. Aus der Perspektive latenter Dispositionen würde man hingegen vermutlich zu einer anderen Einschätzung gelangen, weil es bei Person B Hinweise auf das Vorhandensein relevanter Dispositionen (z. B. Kenntnisse zur Bedeutung von Messwiederholungen) gibt, bei Person A hingegen nicht.

## Forschungsfrage und Hypothese

Die vorausgegangenen Ausführungen zeigen, dass gerade bei der Erfassung von Kompetenzen im Sinne von Dispositionen verschiedene Erhebungsmethoden bei Tests mit Realexperimenten spezifische Vor- und Nachteile aufweisen und sich zudem bezüglich der Testökonomie unterscheiden. Die Wahl einer Methode ist somit immer im Spannungsfeld zwischen Ökonomie und Genauigkeit der Kompetenzerfassung im Sinne von validen Rückschlüssen auf Dispositionen zu treffen. Mit Blick auf diese Genauigkeit der Kompetenzerfassung gibt es nur wenige Studien, welche die Ergebnisse der Kompetenzerfassung anhand verschiedener Erhebungsmethoden systematisch vergleichen. Einige Studien vergleichen beispielsweise die Ergebnisse der Kompetenzerfassung anhand von Schüler*innen-Protokollen und Beobachtungen (bzw. Videoaufnahmen) (z. B. Baxter und Shavelson 1994; Emden und Sumfleth 2012; Schreiber et al. 2016). In der Studie von Hild et al. (2019) werden die Ergebnisse der Kompetenzerfassung anhand von Schüler*innen-Protokollen, Videoaufnahmen mit Lautem Denken und retrospektiven Interviews verglichen. Uns sind jedoch keine Studien bekannt, die systematisch die drei gängigen Erhebungsmethoden Protokolle, Beobachtung und verbale Erläuterungen (z. B. Interviews oder Lautes Denken) vergleichen und somit auch differenzierte Schlüsse diesbezüglich ermöglichen, inwiefern die genutzte Erhebungsmethode die Genauigkeit der Kompetenzerfassung beeinflusst. Solche Erkenntnisse sind jedoch zentral, um je nach Kontext und Ziel der Erfassung der Kompetenzen fundiert entscheiden zu können, welche Erhebungsmethode hinreichend genau und dennoch möglichst ökonomisch ist. Dieses Desiderat greift die vorliegende Studie auf und nimmt dabei exemplarisch Kompetenzen des naturwissenschaftlichen Messens in den Blick, wobei angenommen werden kann, dass die Befunde zum Messen auch Implikationen zur Erfassung von experimentellen Kompetenzen im Allgemeinen zulassen. Es wird davon ausgegangen, dass Schüler*innen-Protokolle eine ökonomische, aber vergleichsweise ungenaue Möglichkeit zur Erfassung der Messkompetenzen darstellen. Ausgehend von dieser Vermutung wird in dieser Studie am Beispiel von Aufgaben mit Realexperimenten zum Messen untersucht, inwiefern durch die *Hinzunahme *weiterer Erhebungsmethoden die Genauigkeit des Ergebnisses der Kompetenzerfassung erhöht werden kann. Hierfür werden die Ergebnisse der Kompetenzerfassung anhand von Schüler*innen-Protokollen (*P*), Schüler*innen-Protokollen und Videoaufnahmen (*PV*), Schüler*innen-Protokollen und Interviews (*PI*) sowie einer Kombination aller drei Methoden (*PVI*) bezüglich der Genauigkeit des Ergebnisses der Kompetenzerfassung verglichen. Ziel ist herauszufinden, welchen Mehrwert verschiedene Kombinationen einzelner Erhebungsmethoden bezüglich der Genauigkeit der Rückschlüsse auf Dispositionen liefern. Die übergeordnete Forschungsfrage lautet:

Inwiefern gibt es bei Aufgaben mit Realexperimenten zum naturwissenschaftlichen Messen systematische Abweichungen zwischen Schüler*innen-Protokollen (P), Schüler*innen-Protokollen und Videos (PV), Schüler*innen-Protokollen und Interviews (PI) sowie einer Kombination aller drei Methoden (PVI) bezüglich der Genauigkeit des Ergebnisses der Kompetenzerfassung?

## Methodisches Vorgehen

### Stichprobe und Studiendesign

Die hier vorgestellte Studie ist eingebettet in das vom Schweizerischen Nationalfonds (SNF) geförderte Projekt „Experimentelle Kompetenzen von 12- bis 15-jährigen Jugendlichen in den Naturwissenschaften (ExKoNawi): Validierung eines interdisziplinären Experimentiertests“, in dessen Rahmen mithilfe einer Gesamtstichprobe von 468 Jugendlichen der Jahrgangsstufe 8 aller Schulniveaus geprüft wurde, inwiefern ein Testinstrument mit Realexperimenten valide Rückschlüsse auf die experimentellen Kompetenzen der Schüler*innen zulässt (Bonetti in Vorbereitung).

Für den im Rahmen vorliegender Studie durchgeführten Vergleich wurden die Kompetenzen von *N* = 27 Schüler*innen des mittleren Leistungsniveaus^1^ erfasst und verglichen (Alter: *M* = 14 Jahre und 1 Monat, *SD* = 5 Monate; 13 Mädchen und 14 Jungen). Die vorliegende Stichprobe bildet einen Teil der Gesamtstichprobe (für einen Überblick vgl. Murer 2023). Die Schüler*innen vorliegender Studie wurden innerhalb eines Zeitraums von zwei bis vier Wochen insgesamt viermal in der Schule besucht, wobei sie bei jedem Besuch eine von sechs Aufgaben mit Realexperiment zum Messen bearbeiteten. Für jede Aufgabe hatten die Schüler*innen 18 min Zeit und experimentierten jeweils allein. Während des Bearbeitens der Aufgaben füllten sie vorstrukturierte Protokolle aus und wurden gleichzeitig videografiert. Nach dem Bearbeiten wurden sie in Einzelinterviews zu ihrem Vorgehen befragt. Da alle Schüler*innen insgesamt vier der sechs Aufgaben zum Messen bearbeiteten, liegen in Summe 108 Schüler*innen-Protokolle, Videoaufnahmen und Interviews für den Vergleich der Ergebnisse der Kompetenzerfassung vor.

### Aufgaben mit Realexperimenten zum naturwissenschaftlichen Messen

**Abb. 1:**
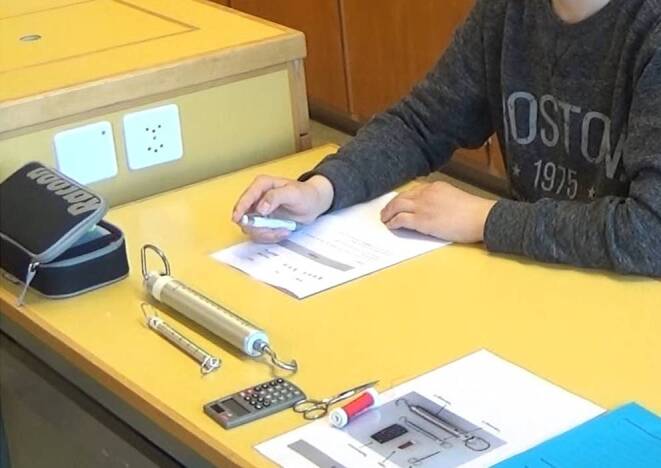
Experimentiermaterial bei der Faden-Aufgabe sowie laminierte Materialkarte und vorstrukturiertes Schüler*innen-Protokoll

Im Rahmen der Studie wurden sechs Aufgaben mit Realexperimenten zum naturwissenschaftlichen Messen verwendet, in denen die Schüler*innen jeweils eine gesuchte Größe möglichst genau messen. Um möglichst genau zu messen, sollen die Schüler*innen das genauere Messinstrument verwenden, Messwiederholungen durchführen und/oder Messunsicherheiten durch Vergrößerung der Menge reduzieren (Metzger und Gut 2017). Bei der Faden-Aufgabe (vgl. auch Metzger et al. 2014) sollten die Schüler*innen zum Beispiel herausfinden, bei welcher Belastung ein Nähfaden reisst. Hierfür bekamen sie einen Faden, eine Schere, zwei verschiedene Federwaagen, die sich in der Genauigkeit ihrer Skalen unterscheiden, einen Taschenrechner sowie eine laminierte Materialkarte mit beschrifteten Experimentiermaterialien (Abb. 1). Anhand von Aufträgen im vorstrukturierten Protokoll wurden die Schüler*innen beispielsweise dazu angeregt, sich zu überlegen, mit welchem Messinstrument sie die Messungen durchführen und wie viele Messwiederholungen sie machen, um ein möglichst genaues Ergebnis zu erhalten. Die Protokolle waren bei allen Aufgaben zum Messen gleich aufgebaut: Zuerst wurde auf einer einführenden Seite die Problemstellung erläutert, anschließend folgten standardisierte Teilaufträge wie zum Beispiel ‚Beschreibe und skizziere, welche Messungen du gemacht hast‘, ‚Mit welchem Messinstrument hast du gemessen?‘, ‚Können beide Messinstrumente gleich genau messen? Begründe‘, ‚Wie viele Male hast du gemessen, bis du dein Ergebnis hattest?‘ oder ‚Wie könntest du genauer messen? Mache Vorschläge.‘^2^

### Datenerhebung

**Abb. 2:**
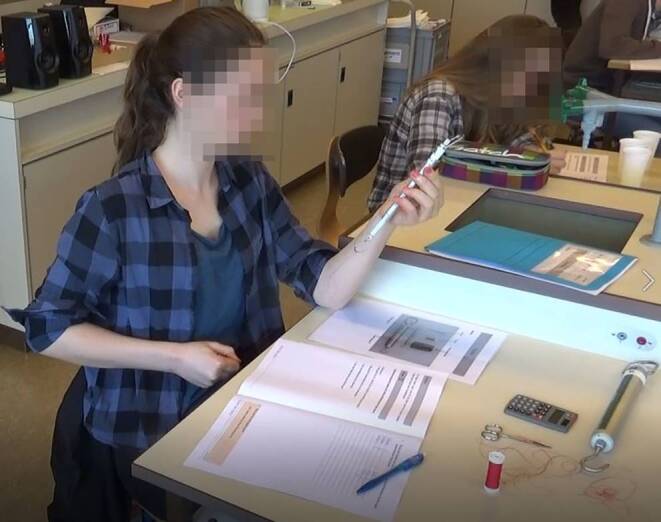
Typische Kameraeinstellung der Videos am Beispiel der Faden-Aufgabe

Während des Bearbeitens der Aufgaben sollten die Schüler*innen ihre Vorgehensweise, Überlegungen, Messwerte, Berechnungen, Ergebnisse und Schlussfolgerungen in vorstrukturierten Protokollen notieren. Gleichzeitig wurden sie auf Video aufgezeichnet. Da die Schüler*innen die Aufgaben in Einzelarbeit lösten und nicht zum Lauten Denken aufgefordert wurden, beinhalten die Videos hauptsächlich experimentelle Handlungen und kaum verbale Äußerungen. Die Kameraeinstellung (Abb. 2) erlaubt das Beobachten von experimentellen Vorgehensweisen (z. B. Durchführen von Messwiederholungen oder Verwendung/Handhabung des Messgeräts), während die Notizen der Schüler*innen (z. B. Messdaten, Berechnungen, Ergebnisse und Begründungen) nicht ersichtlich werden.

Unmittelbar im Anschluss an das Experimentieren wurden die Schüler*innen in Einzelinterviews zu den Experimenten befragt. Die Interviews wurden als Videos aufgezeichnet und dauerten zwischen 11 und 22 min (*M* = 15 min 54 s, *SD* = 2 min 47 s). Für die Interviews standen den Schüler*innen die von ihnen ausgefüllten Protokolle sowie die Experimentiermaterialien der Aufgaben als Stimuli zur Verfügung. Die Interviews wurden in Anlehnung an Helfferich (2011) mit einem Interviewleitfaden geführt. Der Leitfaden umfasste neun Fragen, die sich an den einzelnen Teilaufträge des vorstrukturierten Protokolls orientierten. Falls in diesen Bereichen Notizen der Schüler*innen in den Protokollen vorhanden waren, wurde während des Interviews darauf Bezug genommen. Beispielsweise wurden die Schüler*innen im Interview gefragt ‚Du schreibst, dass du x‑mal die Belastung, bei welcher der Faden reißt, gemessen hast. Wie sind deine Ergebnisse von den Messungen?‘ und ‚Erkläre, wie du dann auf ein Endresultat gekommen bist‘.

### Kodiermanuale

Um Rückschlüsse auf die Dispositionen der Schüler*innen zu ziehen, wurden Kodiermanuale für die Schüler*innen-Protokolle (*P*), die Videoaufnahmen (*V*) und die Interviews (*I*) entwickelt, wobei die Kodiermanuale für die Videos und Interviews aufbauend auf jenen für die Protokolle entwickelt wurden. Ziel der Kodierung war es zu erfassen, wie die Messaufgaben bearbeitet und welche Konzepte zum Messen dabei berücksichtigt wurden. Die Kodiermanuale beinhalten Indikatoren, Ankerbeispiele und gegebenenfalls ergänzende Kodierregeln, wobei sich die Indikatoren in den Kodiermanualen für die Protokolle, Videos und Interviews nur dadurch unterscheiden, dass sie formal für die jeweilige Erhebungsmethode angepasst wurden (z. B. *P*: ‚Die protokollierte Vorgehensweise passt zur Problemstellung‘; *V*: ‚Die im Video ersichtliche Vogehensweise passt zur Problemstellung‘; *I*: ‚Die im Interview beschriebene Vogehensweise passt zur Problemstellung‘). Neben vier Indikatoren, die sich auf die Fähigkeiten zur Durchführung der Messung beziehen und abbilden, ob die Schüler*innen beispielsweise ein geeignetes Messergebnis erzielt haben (z. B. ‚Es ist ein Messwert bzw. Resultat innerhalb der Toleranzbreite vorhanden‘) gibt es elf Indikatoren, die sich auf die Fähigkeiten zur Planung und Auswertung beziehen und die Rückschlüsse auf das Verständnis der zugrundeliegenden Konzepte (K1 bis K5) ermöglichen. In Tab. 1 werden exemplarisch Indikatoren aus dem Kodiermanual der Schüler*innen-Protokolle aufgeführt, welche Hinweise bezüglich der Umsetzung des Konzepts zu ‚Messwiederholung‘ (K4) liefern. Neben den drei Indikatoren für das Konzept ‚Messwiederholung‘ sind in Tab. 1 exemplarische Schüler*innen-Antworten zur Faden-Aufgabe angegeben, die zur Erfüllung des jeweiligen Indikators führen.

**Tab. 1:** Ausschnitt aus dem Kodiermanual der Schüler*innen-Protokolle zum Konzept K4 (Messwiederholungen) und Hinweise zur Kodierung inklusive exemplarischer Schüler*innen-Antworten zur Faden-Aufgabe. Eine vollständige Darstellung ist in Tab. 5 am Ende des Beitrags zu finden

Konzept	Indikator	Beschreibung Indikator	Hinweise für die Kodierung und exemplarische Antworten aus den Schüler*innen-Protokollen (kursiv)
K4: Die Messunsicherheit kann durch das Durchführen von **Messwiederholungen** und anschließender Mittelwertbildung reduziert werden	**MW1**	Es sind Daten zu Messwiederholungen vorhanden. Es wird ein Wert aus der Messreihe als Resultat ausgewählt/berechnet	Im Protokoll wird ersichtlich, dass mehrmals gemessen wurde und ein Wert als Resultat ausgewählt wurde:
			*„1. Messung: 1100* *g, 2. Messung: 1000* *g und 3. Messung 900* *g. Endresultat* *=* *900* *g“. *Es wurde der letzte Wert der Messreihe als Ergebnis ausgewählt
			*„1. Messung: 700* *g, 2. Messung: 900* *g und 3. Messung: 1000* *g. Endresultat* *=* *866,7* *g“. *Es wurde ein Mittelwert berechnet
	**MW2**	Das arithmetische Mittel wurde aus den Werten der Messwiederholungen berechnet	Es wird ersichtlich, dass aus den Werten der Messwiederholungen ein Mittelwert berechnet wurde: *„1. Messung: 700* *g, 2. Messung: 900* *g und 3. Messung: 1000* *g. Endresultat (Mittelwert)* *=* *866,7* *g“*
	**MW3**	Messwiederholungen werden als Lösungsvorschlag zur Steigerung der Messgenauigkeit genannt	Beim Auftrag „Wie könntest du genauer messen? Mache Vorschläge.“ wird auf das Durchführen von Messwiederholungen verwiesen: *„Ich würde noch zweimal mehr messen und dann den Durchschnitt ausrechnen. So würde es noch genauer werden.“*

### Auswertung

Die Schüler*innen-Protokolle, Videoaufnahmen und Interviews wurden zuerst einzeln mit Hilfe der Kodiermanuale ausgewertet. Hierfür wurden die Protokolle, Videos und Interviews separat betrachtet und die Indikatoren als ‚erfüllt‘ (1 Punkt) respektive ‚nicht erfüllt‘ (0 Punkte) beurteilt. Insgesamt konnten 15 Punkte erzielt werden. Es fanden separate Kodierschulungen für die Protokolle, Videos und Interviews statt. Die Schulungen waren inhaltlich analog aufgebaut und umfassten Erläuterungen der Manuale sowie exemplarische Kodierungen von Datensätzen, an denen die Anwendung der Manuale eingeübt wurden. Nach der Kodierschulung wurden die prozentuale Übereinstimmung (pÜ) und die zufallsbereinigte Übereinstimmung mittels Cohens Kappa berechnet. Die gemittelten Übereinstimmungs- beziehungsweise Kappawerte und die zugehörigen Intervalle deuten auf eine zufriedenstellende Interrater-Übereinstimmung hin (*P*: 15 % der Daten doppelt kodiert: Cohens *k* *=* 0,74 [0,61, 1]; pÜ = 93 % [81 %, 100 %]; *V*: 30 % der Daten doppelt kodiert: Cohens *k* = 0,94 [0,77, 1], pÜ = 98 % [91 %, 100 %]; *I*: 30 % der Daten doppelt kodiert: Cohens *k* = 0,84 [0,70, 1], pÜ = 95 % [86 %, 100 %])^3^. Nach der Kodierung auf Basis der einzelnen Erhebungsmethoden wurden die Indikatoren durch die mit Blick auf die Forschungsfrage relevanten Kombinationen (*PV, PI* und *PVI*) ausgewertet. Hierbei galt die Regel, dass sobald ein Indikator in einer Erhebungsmethode als ‚erfüllt‘ beurteilt wurde, dieser auch in der Kombination als ‚erfüllt‘ betrachtet wurde. Wurden zum Beispiel im Schüler*innen-Protokoll keine Messwerte der Messwiederholungen festgehalten, sondern lediglich ein Messergebnis angegeben, wurden für dieses im Protokoll bei den Indikatoren zur Messwiederholung (MW1 und MW2; Tab. 1) null Punkte vergeben. Falls diese Indikatoren im zugehörigen Interview jedoch mit jeweils einem Punkt bewertet wurden, zum Beispiel weil die Äußerungen der Schüler*innen zeigen, dass Messwiederholungen durchgeführt und ein Mittelwert berechnet wurde, dann wurde bei den Kombinationen *PI* und *PVI *jeweils ein Punkt für diese Indikatoren vergeben. Anschließend wurden mithilfe der beurteilten Indikatoren jeweils Summenscores für *P, PV, PI *und *PVI* gebildet. Der Summenscore kann als Maß für das Verständnis der Konzepte K1 bis K5 und damit für die Messkompetenzen der Schüler*innen interpretiert werden; er kann für jede Erhebungsmethode respektive Kombination Werte zwischen 0 und 15 Punkte annehmen. Durch dieses Vorgehen kann die erreichte Punktzahl bei der Hinzunahme weiterer Erhebungsmethoden nur größer und nie kleiner werden, weil sich aus einer größeren Anzahl von Erhebungsmethoden automatisch eine größere Anzahl von Möglichkeiten ergibt, in denen Hinweise bezüglich der Dispositionen von Schüler*innen – hier bezüglich des vorhandenen Verständnisses der Konzepte K1 bis K5 – identifiziert werden können. Eine Stärke dieses Vorgehens besteht darin, dass bei der Kompetenzerfassung der Heterogenität der Schüler*innen Rechnung getragen werden kann. So werden beispielsweise auch Dispositionen von Lernenden sichtbar, wenn sie zwar korrekt handeln (z. B. bei Konzept K2: eine zur Problemstellung passende Vorgehensweise wird im Video ersichtlich), aber Schwierigkeiten dabei haben, Handlungen/Gedanken im Protokoll respektive im Interview zu verbalisieren. Mit dieser Stärke geht jedoch auch eine Herausforderung einher, weil damit auch der Fall auftreten kann, dass eine Erhebungsmethode auf ein angemessenes konzeptuelles Verständnis hindeutet und die Lernenden somit einen Punkt erhalten, obwohl es bei einer andere Methode Hinweise auf ein eher unangemessenes Verständnis gibt. Zum Beispiel gab es drei Fälle, bei welchen anhand der Protokolle davon ausgegangen werden konnte, dass die Schüler*innen das Konzept von Messwiederholungen (K4) verstanden haben (Messwerte wurden protokolliert und ein Mittelwert berechnet). Die Erläuterungen im Interview wiesen dann aber auf ein mangelhaftes Verständnis hin. So gab ein Schüler beispielsweise an, dass er bei der Messwiederholung ein anderes Verfahren genutzt hat und somit keine Messwiederholung im eigentlichen Sinn durchgeführt hat. Solche Fälle, in denen Lernende einen Punkt bekommen, obwohl es auch konkrete Hinweise auf ein nicht vollständig angemessenes konzeptuelles Verständnis gab, wurden insgesamt aber nur sehr selten beobachtet (weniger als 5 % aller bepunkteten Indikatoren), weshalb aus unserer Sicht klar die Vorteile dieses Vorgehens überwiegen.

Durch die gezielten Nachfragen zu den Konzepten in den Interviews und weil Schüler*innen das Protokollieren oft nicht von Anfang an gelingt (z. B. Gott und Duggan 2002; Hild et al. 2019; Emden und Sumfleth 2012), könnte es über die vier Erhebungszeitpunkte zu einem Lerneffekt gekommen sein. Dieser Lerneffekt könnte dazu führen, dass den Schüler*innen beispielsweise das Protokollieren gegen Ende der Erhebung besser gelingt (bzw. dass sie die Protokolle ausführlicher führen), sodass die zu erwartenden Unterschiede in den Ergebnissen der Kompetenzerfassung anhand von *P* und *PI *gegen Ende der Erhebung geringer werden. Um zu untersuchen, ob ein solcher Lerneffekt vorliegt und bei der Deutung der Ergebnisse berücksichtigt werden muss, wurde mit einer Repeated-Measures ANOVA untersucht, inwiefern der Erhebungszeitpunkt einen Effekt auf die Unterschiede der Ergebnisse der Kompetenzerfassung anhand von *P* respektive *PI* hat (abhängige Variable: Differenz im Ergebnis der Kompetenzerfassung anhand von *P* und *PI*; unabhängige Variable: Erhebungszeitpunkt). Daraufhin wurden die Ergebnisse der Kompetenzerfassung sowohl auf Ebene der Stichprobe als auch auf Ebene einzelner Schüler*innen betrachtet und verglichen. Beim *Vergleich auf Ebene der Stichprobe* wurde untersucht, inwiefern die Erhebungsmethoden im Mittel für eine Stichprobe zu ähnlichen Rückschlüssen auf Dispositionen führen. Hierfür wurden Mittelwerte, Mittelwertunterschiede und Streumaße für die verschiedenen Erhebungsmethoden respektive deren Kombinationen berechnet und verglichen. Beim Vergleich auf Ebene einzelner Schüler*innen wurde hingegen untersucht, inwiefern auf individueller Ebene die Erhebungsmethoden zu ähnlichen Rückschlüssen auf Dispositionen führen. Hierfür wurde berechnet, wie stark die Ergebnisse der Kompetenzerfassung durch die Erhebungsmethoden für die einzelnen Schüler*innen miteinander korrelieren. Für einen differenzierten Vergleich der Erhebungsmethoden ist sowohl die Ebene der Stichprobe als auch die Ebene einzelner Schüler*innen relevant (vgl. Schreiber 2012): So kann es beispielsweise sein, dass die Erhebungsmethoden auf Ebene einzelner Schüler*innen *nicht* zu ähnlichen Rückschlüssen bezüglich der Dispositionen führen (niedrige Korrelationen zwischen den Ergebnissen der Kompetenzerfassung), aber auf Ebene der Stichprobe ‚austauschbar‘ sind (geringe Mittelwertunterschiede, *t*‑Test nicht signifikant). Somit wären die unterschiedlichen Ergebnisse der Kompetenzerfassung auf Ebene einzelner Schüler*innen nicht zwangsläufig auf die unterschiedlichen Erhebungsmethoden bei der Kompetenzerfassung zurückzuführen, da sich der Einfluss der unterschiedlichen Erhebungsmethoden auf Ebene der Stichprobe nicht durchzusetzen scheint (vgl. Schreiber 2012). Zudem sagt eine mittelmässige Korrelation auf Ebene einzelner Schüler*innen noch nicht automatisch etwas über die Genauigkeit des Ergebnisses der Kompetenzerfassung aus. Eine mittelmässige Korrelation auf Ebene einzelner Schüler*innen zeigt, dass die Erhebungsmethoden auf dieser Ebene zu ähnlichen Rückschlüssen bezüglich der Dispositionen führen. Es stellt sich jedoch die Frage, inwiefern die Erhebungsmethoden auch auf Ebene der Stichprobe zu vergleichbaren Rückschlüssen gelangen.

## Ergebnisse

Die Ergebnisse der Repated-Measure ANOVA zeigen, dass es keinen Haupteffekt des Erhebungszeitpunkts auf die Differenz im Ergebnis der Kompetenzerfassung anhand von *P* und *PI* gibt (*F* (1,26) = 0,886, *p* = 0,355, *η*^*2*^ = 0,033). Somit kann davon ausgegangen werden, dass sich die Kompetenzen der Lernenden im Verlauf der Erhebung nicht entscheidend verändern.

In der Folge werden die Ergebnisse der Kompetenzerfassung zuerst auf Ebene der Stichprobe und dann auf Ebene einzelner Schüler*innen betrachtet und verglichen. Im Hinblick auf die Forschungsfrage wurde untersucht, inwiefern durch die *Hinzunahme* weiterer Erhebungsmethoden zum Protokoll genauere Rückschlüsse auf die Dispositionen der Schüler*innen ermöglicht werden. Hierfür wurden die Ergebnisse der Kompetenzerfassung anhand von *P* versus *PV *beziehungsweise *P* versus *PI *verglichen. Zusätzlich wurden die Kontraste *PI *versus *PVI* sowie *PV* versus *PVI* angeschaut, um zu prüfen, inwiefern eine Kombination aller drei Erhebungsmethoden zu genaueren Rückschlüssen im Vergleich zur Kombination nur zweier Methoden führt.

### Ergebnisse auf Ebene der Stichprobe

Für die Betrachtung, inwiefern zusätzliche Videoaufnahmen beziehungsweise Interviews auf Ebene der Stichprobe zu ähnlichen Rückschlüssen auf die Dispositionen führen, wurden mittels t‑Tests^4^ Mittelwertunterschiede zwischen den Ergebnissen der Kompetenzerfassung betrachtet (Tab. 2). Die Ergebnisse zeigen, dass sich die Kompetenzwerte durch die Hinzunahme von Videos im Mittel nur geringfügig ändern (*P* vs. *PV*; Tab. 2) oder gar nicht verändern (*PI *vs. *PVI*; Tab. 2). Im Vergleich dazu fallen die mittleren Kompetenzwerte durch die Hinzunahme von Interviews deutlich höher aus als bei einer Kompetenzerfassung allein auf Basis der Protokolle respektive Protokolle und Videos (*P* vs. *PI, PV *vs. *PVI; *Tab. 2). Die Unterschiede entsprechen hier einem mittleren bis großen Effekt (0,5 < *d* < 0,8; Cohen 1988).

**Tab. 2:** Mittelwerte (*M*) und Mittelwertunterschiede der Ergebnisse der Kompetenzerfassung anhand *P*, *PV*, *PI* und *PVI* (0 bis 15 Punkte möglich; *N* = 108)

Mittelwert-Vergleich (*M1 *vs. *M2*)	*M1*	*SD1*	*M2*	*SD2*	*t*	*p*	*d*
P vs. PV	8,30	2,71	8,63	2,48	−5,40	< 0,001	0,13
P vs. PI	8,30	2,71	10,24	2,34	−11,92	< 0,001	0,77
PV vs. PVI	8,63	2,48	10,28	2,32	−12,09	< 0,001	0,69
PI vs. PVI	10,24	2,34	10,28	2,32	−1,42	0,16 (n. s.)	0,02

t‑Test für abhängige Stichproben und Cohens d als Maß für die Effektstärke

Da die mittleren Kompetenzwerte bei der Hinzunahme der Interviews deutlich höher ausfallen als bei der Erfassung ohne Interviews, stellt sich die Frage, ob diese Unterschiede auf einzelne Indikatoren zurückzuführen sind. Falls einzelne Indikatoren als Hauptursache für die Unterschiede identifiziert werden können, lässt sich daraus ableiten, für welche Facetten von Messkompetenzen beziehungsweise für welche Konzepte Schüler*innen-Protokolle womöglich ausreichen und wofür ein zusätzliches Nachfragen, zum Beispiel mit Hilfe von Interviews, einen Mehrwert darstellt. Um dieser Frage nachzugehen, wurden die Mittelwerte der Ergebnisse der Kompetenzerfassung anhand der Schüler*innen-Protokolle (*P*) sowie anhand der Schüler*innen-Protokolle unter Hinzunahme der Interviews (*PI*) deskriptiv auf Indikatorenebene betrachtet. Dabei konnte festgestellt werden, dass sich bei einigen Indikatoren der Konzepte K3 (Messinstrument) und K4 (Messwiederholung) die Mittelwerte von *P* und *PI *deutlich unterscheiden. Im Vergleich dazu zeigte sich, dass sich bei den Indikatoren zu den Konzepten K1 (Messwert und Maßeinheit), K2 (Messungen passen zur Problemstellung) und K5 (Mengenvergrößerung) die Mittelwerte von *P* und *PI nicht* deutlich unterscheiden.^5^

Da sich bei einigen Indikatoren der Konzepte K3 (Messinstrument) und K4 (Messwiederholung) die Mittelwerte der Ergebnisse der Kompetenzerfassung anhand von *P* und *PI* deutlich unterscheiden, werden in Tab. 3 die Mittelwerte dieser Indikatoren deskriptiv aufgeführt (K3: Indikatoren MI1 bis MI3, K4: Indikatoren MW1 bis MW3). Tab. 3 zeigt, dass sich die Mittelwerte von *P* und *PI* vor allem bei den Indikatoren MI1 ‚Das genauere Messinstrument wird verwendet. Die Begründung, warum dieses Messinstrument genauer ist, ist korrekt‘ (*∆M* = 0,34) und MW1 ‚Es sind Daten zu Messwiederholungen vorhanden. Es wird ein Wert aus der Messreihe als Resultat ausgewählt/berechnet‘ (*∆M* = 0,29) deutlich unterscheiden. Eine qualitative Analyse dieser Bereiche ergab, dass in den Schüler*innen-Protokollen oft die Begründungen für die Wahl eines Messinstruments fehlen oder diese nicht ausreichend genau sind. Aufgrund dessen kann anhand der Protokolle nicht beurteilt werden, ob die Schüler*innen das genauere Messinstrument verwendet haben, da sie grundsätzlich das Konzept K3 verstanden haben, oder ob sie das genauere Messgerät aufgrund anderer Aspekte wählen (z. B. eine Federwaage wurde gewählt, da diese besser/einfacher zu bedienen ist). Da es sich bei der Begründung zur Wahl eines Messinstruments um einen kognitiven Prozess handelt, wird dieser Aspekt zudem nicht in den Videoaufnahmen ersichtlich und die Erkenntnis, ob Indikator MI1 erfüllt ist, kann somit oft erst in den Interviews gewonnen werden. Zudem zeigt die qualitative Analyse im Bereich des Indikators MW1, dass die Schüler*innen oft die Messwerte der Messwiederholungen nicht protokollieren, sondern lediglich ein Schlussresultat angeben (z. B. ‚Der Faden reißt bei einer Belastung von 800 g.‘). Somit wird anhand der Protokolle nicht ersichtlich, dass Messwiederholungen durchgeführt wurden, dann aber nur ein Ergebnis protokolliert wurde. Während das Durchführen von Messwiederholungen zwar in den Videoaufnahmen ersichtlich wird, kann der Umgang mit den aufgenommenen Messwerten (z. B. Berechnung des arithmetischen Mittels) anhand der Videos nicht beurteilt werden. Da der Umgang mit den aufgenommenen Messwerten jedoch Bestandteil von Indikator MW1 ist, kann dieser Indikator somit oft erst anhand der Interviews beurteilt werden (beispielhafte Schüler*innen-Aussage im Interview: ‚Ich habe mehrmals gemessen und der Faden ist immer bei einer Belastung zwischen 700 und 900 g gerissen. Darum habe ich als Schlussresultat 800 g angegeben.‘).

**Tab. 3:** Deskriptive Betrachtung der Mittelwerte der Kompetenzerfassung anhand von *P* und *PI* bei den Indikatoren im Bereich der Konzepte K3 ‚Wahl des Messinstruments‘ (MI1 bis MI3) und K4 ‚Messwiederholung‘ (MW1 bis MW3). Die Indikatoren wurden mit ‚erfüllt‘ (1 Punkt) respektive ‚nicht erfüllt‘ (0 Punkte) beurteilt. *N* = 108

Konzept/Indikator	*P*		*PI*		*∆M*
	*M*	*SD*	*M*	*SD*	
*K3: Je höher die Genauigkeit des verwendeten Messinstruments ist, desto geringer ist die Messunsicherheit*					
MI1: Das genauere Messinstrument wird verwendet. Die Begründung, warum dieses Messinstrument genauer ist, ist korrekt	0,52	0,50	0,86	0,35	0,34
MI2: Es wird deutlich, dass *für die Lösung *mit dem genaueren Messinstrument gemessen wurde	0,85	0,36	0,94	0,25	0,09
MI3: Das Messen mit einem (noch) genaueren Messinstrument wird als Lösungsvorschlag zur Steigerung der Messgenauigkeit angegeben	0,15	0,36	0,30	0,46	0,15
*K4: Die Messunsicherheit kann durch das Durchführen von Messwiederholungen und anschlieβender Mittelwertbildung reduziert werden*					
MW1: Es sind Daten zu Messwiederholungen vorhanden. Es wird ein Wert aus der Messreihe als Resultat ausgewählt/berechnet	0,55	0,50	0,84	0,37	0,29
MW2: Das arithmetische Mittel wurde aus den Werten der Messwiederholungen berechnet	0,41	0,49	0,52	0,50	0,11
MW3: Messwiederholungen werden als Lösungsvorschlag zur Steigerung der Messgenauigkeit genannt	0,15	0,36	0,32	0,47	0,17

Um den Einfluss der beiden mit Blick auf die Unterschiede zwischen *P* und *PI* besonders relevanten Indikatoren genauer einordnen zu können, wurde ein weiterer Vergleich durchgeführt, bei dem die beiden Indikatoren MI1 und MW1 aus der Analyse ausgeschlossen und die Kontraste der mittleren Kompetenzwerte von *P* und *PI* erneut berechnet wurden. Dabei konnte festgestellt werden, dass auch nach Ausschluss dieser beiden Indikatoren die mittleren Kompetenzwerte von *PI* signifikant höher sind als diejenigen von *P *(*M*_*P*_ = 7,23, *SD*_*P*_ = 2,35, *M*_*PI*_ = 8,54, *SD*_*PI*_ = 2,09, *t* = −9,44, *p* < 0,001, *d* = 0,59, *N* = 108). Zwar fällt der Effekt des Mittelwertunterschieds nach Ausschluss der beiden Indikatoren geringer aus (*d* = 0,59) im Vergleich zum Effekt ohne Ausschluss der Indikatoren (*d* = 0,77; Tab. 2), dennoch entspricht der Mittelwertunterschied immer noch einem mittleren Effekt (vgl. Cohen 1988) und ist somit als bedeutsam einzustufen. Zusätzliche Interviews führen also auch unter Ausschluss dieser beiden Indikatoren im Mittel zu höheren Ergebnissen der Kompetenzerfassung, weshalb davon auszugehen ist, dass diese Indikatoren nicht allein für die beobachteten Mittelwertunterschiede verantwortlich sind.

### Ergebnisse auf Ebene einzelner Schüler*innen

Für die Betrachtung, inwiefern zusätzliche Videoaufnahmen beziehungsweise Interviews auf der Ebene einzelner Schüler*innen zu ähnlichen Rückschlüssen auf die Dispositionen führen, wurden die Korrelationen der Ergebnisse der Kompetenzerfassung betrachtet (Tab. 4). Dabei wurden die Korrelationen sowohl unter Berücksichtigung aller Indikatoren als auch unter Ausschluss der Indikatoren MI1 und MW1 betrachtet, da bei diesen beiden Indikatoren auf Ebene der Stichprobe besonders große Unterschiede zwischen den Ergebnissen der Kompetenzerfassung anhand von *P* und *PI *festgestellt werden konnten. Durch den Vergleich dieser beiden Betrachtungen sollen Erkenntnisse darüber gewonnen werden, welchen Einfluss die Indikatoren MI1 und MW1 auf Ebene einzelner Schüler*innen auf das Ergebnis der Kompetenzerfassung haben.

**Tab. 4:** Korrelationen zwischen den Ergebnissen der Kompetenzerfassung anhand von *P*, *PV*, *PI *und *PVI *unter Berücksichtigung aller Indikatoren und unter Ausschluss der Indikatoren MI1 und MW1 (*N* = 108)

Korrelationen unter Berücksichtigung aller Indikatoren		Korrelationen unter Ausschluss der Indikatoren MI1 und MW1	
*P* und *PV*:	*r* = 0,94; *p* ≤ 0,001	*P* und *PV*:	*r* = 0,92; *p* ≤ 0,001
*P* und *PI*:	*r* = 0,67; *p* ≤ 0,001	*P* und *PI*:	*r* = 0,70; *p* ≤ 0,001
*PV* und *PVI*:	*r* = 0,71; *p* ≤ 0,001	*PV* und *PVI*:	*r* = 0,74; *p* ≤ 0,001
*PI* und *PVI*:	*r* = 0,99; *p* ≤ 0,001	*PI* und *PVI*:	*r* = 0,99; *p* ≤ 0,001

Es wurde der Korrelationskoeffizient Kendall-Tau‑b verwendet

Tab. 4 zeigt, dass die Korrelationen zwischen den Ergebnissen der Kompetenzerfassung anhand von *P* und *PV* (*r* = 0,94 bzw. *r* = 0,92) sowie anhand von *PI* und *PVI* (*r* = 0,99) sehr hoch ausfallen. Diese Erhebungsmethoden kommen also zu sehr ähnlichen Ergebnissen der Kompetenzerfassung. Im Gegensatz dazu kann festgestellt werden, dass die Ergebnisse der Kompetenzerfassung anhand von *P* und *PI* (*r* = 0,67 bzw. *r* = 0,70) sowie *PV* und *PVI *(*r* = 0,71 bzw. *r* = 0,74), auch unter Ausschluss der Indikatoren MI1 und MW1, nur mittelmäßig hoch miteinander korrelieren.

## Diskussion und Implikation

Realexperimente sind ein etabliertes und wichtiges Verfahren zur Erfassung experimenteller Kompetenzen. Grundsätzlich können die experimentellen Kompetenzen bei Tests mit Realexperimenten mit unterschiedlichen Erhebungsmethoden erfasst werden, insbesondere Schüler*innen-Protokolle (*P*), Videoaufnahmen (*V*) und Interviews (*I*) werden häufig genutzt, wobei die Wahl einer Methode stets im Spannungsfeld zwischen Ökonomie und Genauigkeit der Kompetenzerfassung zu treffen ist. Im vorliegenden Beitrag wurde am Beispiel des naturwissenschaftlichen Messens als einer wichtigen Facette experimenteller Kompetenzen untersucht, inwiefern ergänzende Videos respektive Interviews zu Schüler*innen-Protokollen genauere Rückschlüsse auf Dispositionen von Schüler*innen ermöglichen.

Die Ergebnisse der Studie zeigen, dass sich die Mittelwerte der Kompetenzwerte zwischen Erhebungsmethoden mit und ohne zusätzliche Interviews auf Ebene der Stichprobe signifikant und bedeutsam unterscheiden. Zudem sind die Korrelationen der Kompetenzwerte einzelner Schüler*innen zwischen den Erhebungsmethoden mit und ohne zusätzliche Interviews nur mittelmäßig hoch. Zusammen deuten die Ergebnisse darauf hin, dass Erhebungsmethoden mit und ohne zusätzliche Interviews bezüglich der Genauigkeit der Rückschlüsse auf Dispositionen nicht austauschbar sind und zusätzliche Interviews zu genaueren Ergebnissen der Kompetenzerfassung führen (*PI* genauer als *P*; *PVI *genauer als *PV*). Diese Erkenntnis stützt Ergebnisse anderer Studien, die zeigen, dass Schüler*innen-Protokolle allein nur eine eingeschränkt genaue Kompetenzerfassung ermöglichen (vgl. z. B. Abrahams et al. 2013; Gott und Duggan 2002; Gut-Glanzmann 2012; Hild et al. 2019). Ein wesentlicher Unterschied zwischen Erhebungsmethoden mit und ohne zusätzliche Interviews ist, dass Schüler*innen zum Teil nicht alles in den Protokollen dokumentieren können (z. B. aufgrund fehlender sprachlicher Fähigkeiten) oder wollen (z. B. weil es viel Arbeit macht) und so nicht vollständig ersichtlich wird, was sie tatsächlich getan und gedacht haben (vgl. z. B. auch Gott und Duggan 2002; Gut-Glanzmann 2012; Hild et al. 2019). Entsprechend lassen Schüler*innen-Protokolle nur eine eingeschränkt genaue Aussage über die Dispositionen von Schüler*innen zu, insbesondere bei Schüler*innen mit niedrigen sprachlichen Fähigkeiten oder geringer Motivation.

Die Befunde der vorliegenden Studie zeigen zudem, dass die Mittelwertunterschiede der Kompetenzwerte zwischen Erhebungsmethoden mit und ohne zusätzliche Videos auf Ebene der Stichprobe einem kleinen Effekt entsprechen (*P* vs. *PV*) oder nicht signifikant sind (*PV* vs. *PVI*). Auf Ebene einzelner Schüler*innen wurden zudem signifikante und hohe Korrelationen zwischen *P* und *PV* sowie *PI *und *PVI* beobachtet. In Summe deuten die Ergebnisse der Studie somit darauf hin, dass zusätzliche Videos keinen entscheidenden Mehrwert bezüglich der Genauigkeit der Ergebnisse der Kompetenzerfassung liefern, insbesondere dann nicht, wenn zusätzliche Interviews durchgeführt wurden (*PI* ≈ *PVI*). Dies kann vermutlich damit erklärt werden, dass bei der vorliegenden Studie die Schüler*innen während des Experimentierens nicht zum Lauten Denken aufgefordert wurden und somit in den Videoaufnahmen hauptsächlich experimentelle Handlungen und keine kognitiven Prozesse ersichtlich werden. Dies führt dazu, dass anhand der Videos oft nicht beurteilt werden kann, ob die Schüler*innen das zugrundeliegende Konzept grundsätzlich verstanden haben. Beispielsweise kann anhand der Videos zwar beobachtet werden, ob Messwiederholungen durchgeführt wurden, inwiefern die Lernenden jedoch das Konzept zu Messwiederholungen verstanden haben und sie deshalb zur Reduktion der Messunsicherheit durchführen und nicht aufgrund anderer Aspekte (z. B. Messwiederholungen wurden durchgeführt, da es bei der ersten Messung Schwierigkeiten gab), wird jedoch oft erst anhand zusätzlicher Interviews deutlich. Dieser Befund steht auf den ersten Blick im Widerspruch zu Arbeiten von Hild et al. (2019), die zum Ergebnis kamen, dass Beobachtungen respektive Videoaufnahmen zu einem genaueren Ergebnis der Kompetenzerfassung führen. Bei genauerer Betrachtung zeigt sich jedoch, dass die Schüler*innen bei Hild und anderen im Rahmen der Videoaufzeichnungen auch dazu aufgefordert wurden, ihre Gedanken zu verbalisieren. Wir nehmen deshalb an, dass der von Hild und anderen beobachtete Mehrwert von Videoaufnahmen primär auf das Verbalisieren (vgl. Ergebnisse dieser Studie zu zusätzlichen Interviews) und nicht auf die Videoaufzeichnungen selbst zurückzuführen ist.

Die detaillierte Analyse der Unterschiede zwischen Erhebungsmethoden mit und ohne zusätzliche Interviews zeigt, dass insbesondere die Umsetzung und das Verständnis von Konzepten zur Wahl eines geeigneten Messinstruments (K3) und zu Messwiederholungen (K4) nur mit Protokollen und Videos aber ohne zusätzliche Interviews weniger genau erfasst werden. Während sich bezüglich K3 und K4 relevante Unterschiede finden, zeigen die Ergebnisse auch, dass für ausgewählte Facetten von Messkompetenzen Schüler*innen-Protokolle und allenfalls ergänzende Videos bereits relativ genaue Rückschlüsse bezüglich der Dispositionen von Schüler*innen ermöglichen. Beispielsweise lässt sich die Fähigkeit zur Messdurchführung (Messwert bzw. Ergebnis im Toleranzbereich) sowie die Umsetzung und das Verständnis der Konzepte K1 (Messwerte mit korrekter Maßeinheit) und K2 (Messungen passen zur Problemstellung) relativ genau anhand von Schüler*innen-Protokollen erfassen, wobei mit Blick auf K2 zusätzliche Videos die Genauigkeit der Kompetenzerfassung leicht erhöhen. Auch im Bereich des Konzepts Mengenvergrößerung (K5) scheinen Schüler*innen-Protokolle ziemlich genaue Rückschlüsse auf die Dispositionen von Schüler*innen zu liefern. Während sich bei der genauen Betrachtung der Indikatoren zum Konzept Messwiederholung (K4) herausstellte, dass die Schüler*innen die Messwerte der Messwiederholung oft nicht in den Protokollen dokumentieren, scheint diese Schwierigkeit beim Konzept Mengenvergrößerung weniger aufzutreten: Die Schüler*innen halten meistens den Messwert für eine Menge sowie die Berechnung, anhand der sie diesen Wert auf die gesuchte Grösse zurückrechnen, in den Protokollen fest. Zusammenfassend lässt sich aber festhalten, dass zusätzliche Interviews zu den Schüler*innen-Protokollen genauere Rückschlüsse bezüglich der Dispositionen von Schüler*innen ermöglichen und daraus beispielsweise auch präzisere Hinweise zur Förderung dieser Kompetenzen abgeleitet werden können. Somit scheint es ertragreich, die Schüler*innen nach ihren Vorgehensweisen und Überlegungen beim Experimentieren zu befragen, insbesondere dann, wenn im Zuge der Kompetenzerfassung auch auf das Verständnis der Konzepte zur Wahl eines geeigneten Messinstruments (K3) und zu Messwiederholungen (K4) geschlossen werden soll.

### Limitationen und Ansatzpunkte für zukünftige Forschungsarbeiten

Bei der Interpretation der Ergebnisse der Studie ist zu bedenken, dass die verglichenen Erhebungsmethoden zum Teil etwas unterschiedliche Fähigkeiten erfassen. Damit ein Indikator zum Konzept Messwiederholungen als erfüllt gilt, müssen die Schüler*innen unter anderem im Protokoll und im Video Messwiederholungen *anwenden*, während sie im Interview hierzu ihre Vorgehensweisen *erläutern*. Da zur Entfaltung dieser zum Teil etwas unterschiedlichen Fähigkeiten jedoch die gleichen Konzepte (intuitiv) verstanden sein müssen (hier z. B. K4), erscheint uns der Vergleich der Erhebungsmethoden dennoch sinnvoll und ertragreich. Zweifelsfrei sollten Schüler*innen nicht nur nach einem Experiment richtig erläutern können, was man eigentlich hätte tun müssen (z. B. Messwiederholungen durchführen), sondern auch in der Experimentiersituation selbst entsprechend handeln. Für die Planung der nächsten Schritte zur Unterstützung des Kompetenzaufbaus ist es aber wichtig, genau zu verstehen, ob die Schüler*innen zum Beispiel das Konzept der Messwiederholung selbst noch nicht hinreichend verstanden haben oder ob das Ausbleiben von Messwiederholungen beim Experimentieren auf andere Ursachen zurückzuführen ist.

Eine weitere zentrale Limitation der Studie besteht darin, dass der Vergleich der Erhebungsmethoden mit Schüler*innen des mittleren Leistungsniveaus durchgeführt wurde. Es bleibt somit unklar, inwiefern sich die Ergebnisse auch auf leistungsstärkere Schüler*innen, denen womöglich das Führen eines Protokolls besser gelingt, übertragen lassen. Umgekehrt stellt sich auch die Fragen, ob bei leistungsschwächeren Schüler*innen zusätzliche Videos möglicherweise doch einen Mehrwert bezüglich der Genauigkeit der Rückschlüsse auf Dispositionen bringen, da für sie das Verbalisieren in Protokollen oder Interviews womöglich noch anspruchsvoller ist und mit einer kognitiven Überlastung einhergehen könnte (vgl. Emden und Sumfleth 2012).

Abschließend ist festzuhalten, dass die Erhebungsmethoden Protokoll, Interview und Video in dieser Studie exemplarisch am Beispiel des Messens verglichen wurden. Wir gehen davon aus, dass sich die Überlegungen und Befunde auch auf andere Facetten experimenteller Kompetenzen übertragen lassen, weil beispielsweise auch beim ‚Vergleichen‘ oder ‚Untersuchen‘ Messungen durchgeführt werden müssen und anhand der Vorgehensweise der Schüler*innen auf ihr Verständnis bezüglich zentraler Konzepte (z. B. fairer Vergleich oder Variablenkontrollstrategie) geschlossen werden kann. Somit kann zum Beispiel auch beim ‚Vergleichen‘ oder ‚Untersuchen‘ angenommen werden, dass das Verständnis bezüglich solcher Konzepte in den Schüler*innen-Protokollen und Videos nicht ausreichend ersichtlich wird und somit Interviews genauere Rückschlüsse auf die Dispositionen zulassen. Eine empirische Prüfung der Übertragbarkeit vorliegender Befunde auf weitere Facetten experimenteller Kompetenzen steht jedoch noch aus.

## Anhang

**Tab. 5:** Kodiermanual der Schüler*innen-Protokolle und Hinweise zur Kodierung inklusive exemplarischer Schüler*innen-Antworten zur Faden-Aufgabe

Konzept	Indikator	Beschreibung Indikator	Hinweise für die Kodierung und *exemplarische Antworten aus den Schüler*innen-Protokollen (kursiv)* Die hier aufgeführten Antworten kamen in ähnlicher Form in den Schüler*innen-Protokollen vor. Sie wurden für vorliegende Tabelle leicht umformuliert bzw. zusammengefasst
**Allgemeine Indikatoren**, die sich auf die Fähigkeiten zur Durchführung der Messung beziehen	**AI1**	Die Messung kann durchgeführt werden, sodass mind. ein Messwert innerhalb der definierten Toleranzbreite liegt. Definierte Toleranzbreite: 1 Faden: 600–1400 g; 2 Fäden: 1400–2800 g	Es wird ersichtlich, dass die Messung durchgeführt werden konnte und mind. ein Messwert innerhalb der Toleranzbreite resultierte: *„Ich habe mit Federwaage A gemessen. Mit einem Faden reißt es bei ungefähr 1000* *g.“*
	**AI2**	Es wurde mind. einmal das genauere Messinstrument verwendet	Im Protokoll wird deutlich, dass mind. einmal das genauere Messinstrument (Federwaage A) verwendet wurde: *„Ich habe mit Federwaage A gemessen. Mit einem Faden reißt es bei ungefähr 1000* *g.“*
	**AI3**	Die Messwerte passen zur protokollierten Vorgehensweise	Die Messwerte passen zur protokollierten Vorgehensweise (1 Faden: Werte zwischen 600 und 1400 g; 2 Fäden: Werte zwischen 1400 und 2800 g):
			*„Ich habe den Faden an die Federwaage geknotet und am anderen Ende gezogen, bis es gerissen ist. Der Faden reißt bei ungefähr 1000* *g.“*
			*„Ich habe ein Stück Faden genommen und die Enden zusammengebunden. Die Schlaufe habe ich an die Federwaage gehängt und dann gezogen, bis es gerissen ist. Es reißt bei ungefähr 2550* *g.“*
	**AI4**	Es ist ein *eindeutiges Resultat* innerhalb der Toleranzbereite vorhanden. Definierte Toleranzbreite: 1 Faden: 600–1400 g; 2 Fäden: 1400–2800 g	Es muss ersichtlich werden, dass es ein eindeutiges Resultat ist (z. B. doppelt unterstrichener Wert; Endergebnis einer Rechnung gekennzeichnet mit Gleichheitszeichen; etc.): *„Ich habe den Faden an der Federwaage angemacht und gezogen, bis er gerissen ist. Beim ersten Mal ist er bei 1000* *g gerissen, beim zweiten Mal bei 1100* *g und beim dritten Mal bei 900* *g. Endergebnis* *=* *900* *g“*
K1: Ein Messwert muss immer mit der zugehörigen **Maßeinheit** angegeben werden	**ME1**	Das eindeutige Resultat hat die richtige Einheit	*„Der Faden ist bei 1000* *g gerissen.“*
K2: Eine geplante Messung muss zur zugrundeliegenden Problemstellung passen (**Messplanung**)	**MP1**	Die protokollierte Vorgehensweise passt zur Problemstellung	Anhand einer Beschreibung oder anhand von Skizzen wird eine Vorgehensweise ersichtlich, welche zur Problemstellung passt:
			*„Ich habe den Faden an die Federwaage geknotet und am anderen Ende gezogen, bis es gerissen ist.“*
			*Skizze, die eine adäquate Vorgehensweise zeigt:*
			*„Ich habe ein Stück Faden genommen und die Enden zusammengebunden. Die Schlaufe habe ich an die Federwaage gehängt und dann gezogen, bis es gerissen ist.“*
			*Skizze, die eine adäquate Vorgehensweise zeigt:*
K3: Je höher die Genauigkeit des verwendeten **Messinstruments** ist, desto geringer ist die Messunsicherheit	**MI1**	Das genauere Messinstrument wird verwendet. Die Begründung, warum dieses Messinstrument genauer ist, ist korrekt	Im Protokoll wird ersichtlich, dass mit dem genaueren Messinstrument (Federwaage A) gemessen wurde. Zudem steht im Protokoll eine korrekte Begründung, warum Federwaage A genauer ist: *„Ich habe mit Federwaage A gemessen, weil diese genauer ist. Bei der Federwaage A ist die Skala in 20* *g‑Schritten und bei der Federwaage B in 100* *g‑Schritten.“*
	**MI2**	Es wird deutlich, dass für *die Lösung* mit dem genaueren Messinstrument gemessen wurde	*„Ich habe Federwaage A genommen, weil sie genauer ist. Daraufhin habe ich den Faden mit einem Knoten an der Federwaage angebunden und dann am Faden gezogen, bis er gerissen ist. Das habe ich dreimal gemacht und dann den Durchschnitt berechnet.“*
	**MI3**	Das Messen mit einem (noch) genaueren Messinstrument wird als Lösungsvorschlag zur Steigerung der Messgenauigkeit angegeben	Beim Auftrag „Wie könntest du genauer messen? Mache Vorschläge.“ wird auf ein genaueres Messinstrument verwiesen: *„Ich würde mit einer automatischen/digitalen Federwaage messen. So kann man es besser ablesen.“*
K4: Die Messunsicherheit kann durch das Durchführen von **Messwiederholungen** und anschließender Mittelwertbildung reduziert werden	**MW1**	Es sind Daten zu Messwiederholungen vorhanden. Es wird ein Wert aus der Messreihe als Resultat ausgewählt/berechnet	Im Protokoll wird ersichtlich, dass mehrmals gemessen wurde und ein Wert als Resultat ausgewählt wurde:
			*„1. Messung: 1100* *g, 2. Messung: 1000* *g und 3. Messung 900* *g.* *Endresultat* *=* *900* *g“. *Es wurde der letzte Wert der Messreihe als Ergebnis ausgewählt
			*„1. Messung: 700* *g, 2. Messung: 900* *g und 3. Messung: 1000* *g.* *Endresultat* *=* *866,7* *g“. *Es wurde ein Mittelwert berechnet
	**MW2**	Das arithmetische Mittel wurde aus den Werten der Messwiederholungen berechnet	Es wird ersichtlich, dass aus den Werten der Messwiederholungen ein Mittelwert berechnet wurde: *„1. Messung: 700* *g, 2. Messung: 900* *g und 3. Messung: 1000* *g. Endresultat (Mittelwert)* *=* *866,7* *g“*
	**MW3**	Messwiederholungen werden als Lösungsvorschlag zur Steigerung der Messgenauigkeit genannt	Beim Auftrag „Wie könntest du genauer messen? Mache Vorschläge.“ wird auf das Durchführen von Messwiederholungen verwiesen: *„Ich würde noch zweimal mehr messen und dann den Durchschnitt ausrechnen. So würde es noch genauer werden.“*
K5: Die Messunsicherheit kann durch Vergrößerung der Menge, mit der gemessen wird, reduziert werden (**Mengenvergrösserung**)	**MV1**	Es ist ein Messwert für eine Menge vorhanden	In der Skizze oder dem beschriebenen Versuchsaufbau wird ersichtlich, dass mit doppeltem Faden (oder x‑fachem Faden) gemessen wurde. Für die Messung mit bspw. doppeltem Faden ist ein Messwert vorhanden: *„Ich habe mit doppeltem Faden gemessen. Der Faden riss bei einer Belastung von 1600* *g.“*
	**MV2**	Der Wert der Menge wurde auf die gesuchte Größe zurückgerechnet	*„Bei der Messung mit doppeltem Faden riss der Faden bei 1600* *g. Würde ich nur mit einem Faden messen, würde er schon früher reißen, vermutlich bei 800* *g.“*
	**MV3**	Das Messen mithilfe einer Vergrößerung der Menge wird als Lösungsvorschlag zur Steigerung der Messgenauigkeit vorgeschlagen	Beim Auftrag „Wie könntest du genauer messen? Mache Vorschläge.“ wird z. B. auf das Messen mit doppelten Faden verwiesen: *„Ich würde mit doppeltem Faden messen, weil mit einem Faden geht es sehr schnell, bis der Faden reißt, man kann es kaum ablesen.“*

## Footnotes

1 Jugendliche der Sekundarstufe A (gesamtes Niveauspektrum: Gymnasium, Sekundarstufe A, B und C; wobei das Niveau A das leistungsstärkste Niveau der Sekundarstufe ist).

2 Bei der Formulierung der Aufträge wird von einer „Erhöhung der Messgenauigkeit“ anstelle von einer „Reduktion der Messunsicherheiten“ gesprochen, da ersteres näher am alltäglichen Sprachgebrauch der Schüler*innen ist und somit besser verstanden wird.

3 Die Anteile der Doppelkodierung unterscheiden sich, da die Schüler*innen-Protokolle im Rahmen der Gesamtstudie (*N* = 468), die Videos und Interviews (*N* = 27) im Rahmen vorliegender Studie kodiert wurden. Die Daten in der Klammer zeigen die Mittelwerte und Intervalle der Übereinstimmung. Beispielsweise liegt bei den Protokollen der gemittelte Kappa-Wert bei 0,74, das minimale Kappa bei 0,61 und das maximale bei 1. Die Daten der prozentualen Übereinstimmung (pÜ) sind entsprechend zu interpretieren. Dass sich die Übereinstimmungsmaße der Schüler*innen-Protokolle, Videos und Interviews unterscheiden, ist vermutlich gröβtenteils auf die Eigenschaften des unterschiedlichen Datenmaterials zurückzuführen. Indikatoren, welche in den Videos ersichtlich werden (z. B. es wurde mit einer größeren Menge gemessen) sind oft relativ eindeutig einzuschätzen. Gleiches gilt auch für die Indikatoren der Interviews, da die Schüler*innen ihre Vorgehensweisen in den Interviews meist ziemlich ausführlich erklären. Die Einschätzung von Indikatoren anhand von Schüler*innen-Protokollen fällt im Vergleich dazu oft schwerer, da die Notizen der Schüler*innen allenfalls interpretiert werden müssen.

4 Die Daten wurden mittels Shapiro-Wilk-Test auf Normalverteilung geprüft. Es zeigte sich, dass die Daten nicht normalverteilt, sondern leicht linksschief verteilt sind. Da im Allgemeinen angenommen werden kann, dass t‑Tests robust gegenüber einer Verletzung der Normalverteilung sind (Rasch und Guiard 2004) und ein Wechsel auf nicht-parametrische Tests mit einem deutlich Informationsverlust einhergeht, wurden dennoch t‑Tests für die Analysen genutzt.

5 Für eine ausführliche Darstellung der Ergebnisse siehe Murer (2023).
